# Correction: Accumulation and expression of horizontally acquired genes in *Arcobacter cryaerophilus* that thrives in sewage

**DOI:** 10.7717/peerj.3269/correction-1

**Published:** 2018-10-01

**Authors:** Jess A. Millar, Rahul Raghavan

**Affiliations:** Biology Department, Portland State University, Portland, OR, United States

Corrigendum:

Due to uncertainty associated with the identities of the putative antibiotic resistance genes (ARGs), the authors have edited the article’s title and edited the text to focus more generally on the large number of horizontally acquired genes present in Arcobacter cryaerophilus’s genome. The title is now “Accumulation and expression of horizontally acquired genes in Arcobacter cryaerophilus that thrives in sewage”.

The authors have also clarified the methodology used to identify potential ARGs and have added a new table (Table S7) that includes the E-value, percent identity and percent coverage for each putative ARG that was detected within the contigs.

