# Supplementary material for: Accumulation and expression of multiple antibiotic resistance genes in Arcobacter cryaerophilus that thrives in sewage
Source: PeerJ. 2017 Apr 25;5:e3269. doi: 10.7717/peerj.3269 (PMC5407278; doi:10.7717/peerj.3269)
Supplement: Supplemental Information 1 [file peerj-05-3269-s001.pdf]

FIG S1. *Arcobacter* housekeeping gene trees. Bayesian phylogenic trees for nucleotide sequences of *Arcobacter* housekeeping genes **(A)** *atpA* (618nt) **(B)** *gyrA* (684nt) **(C)** *hsp60* (285nt) and **(D)** *rpoB* (618nt) to identify sewage contigs belonging to *A. cryaerophilus* (in blue).

A

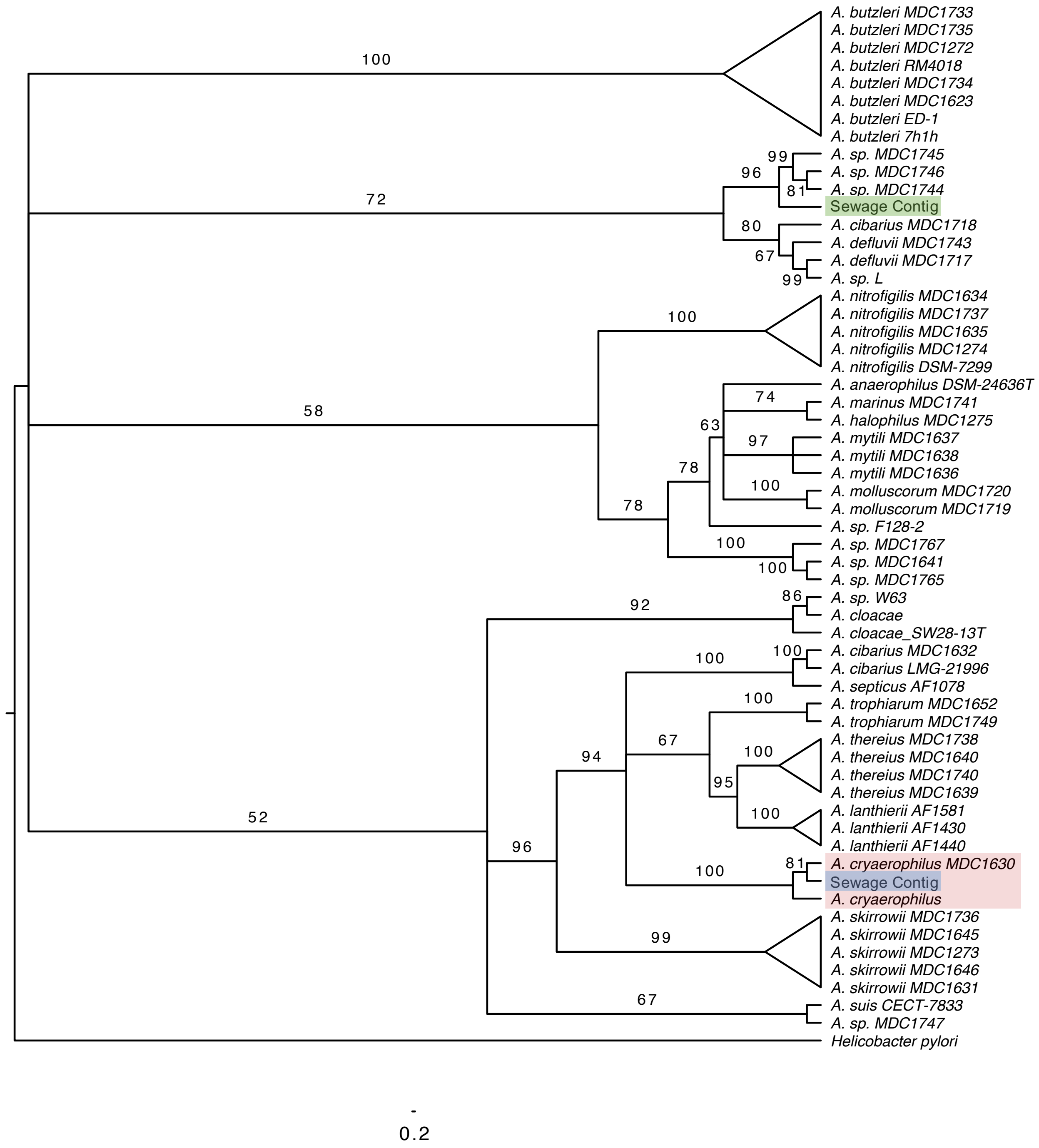

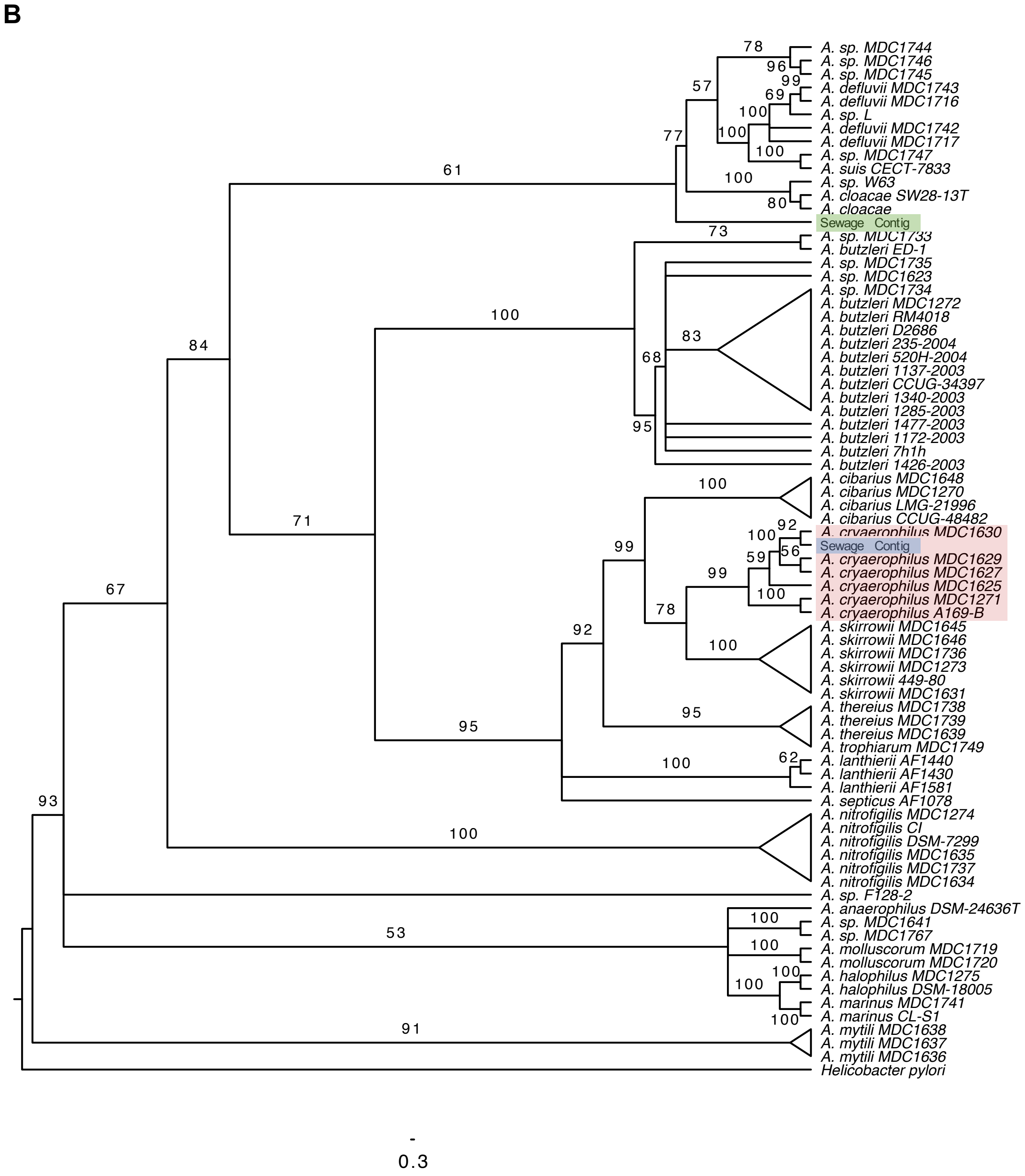
B
A. sp. MDC1744
A. sp. MDC1746
A. sp. MDC1745
A. defluvii MDC1743
A. defluvii MDC1716
A. sp. L
A. defluvii MDC1742
A. defluvii MDC1717
A. sp. MDC1747
A. suis CECT-7833
A. sp. W63
A. cloacae SW28-13T
A. cloacae
Sewage Contig
A. sp. MDC1733
A. butzleri ED-1
A. sp. MDC1735
A. sp. MDC1623
A. sp. MDC1734
A. butzleri MDC1272
A. butzleri RM4018
A. butzleri D2686
A. butzleri 235-2004
A. butzleri 520H-2004
A. butzleri 1137-2003
A. butzleri CCUG-34397
A. butzleri 1340-2003
A. butzleri 1285-2003
A. butzleri 1477-2003
A. butzleri 1172-2003
A. butzleri 7h1h
A. butzleri 1426-2003
A. cibarius MDC1648
A. cibarius MDC1270
A. cibarius LMG-21996
A. cibarius CCUG-48482
A. cryaerophilus MDC1630
Sewage Contig
A. cryaerophilus MDC1629
A. cryaerophilus MDC1627
A. cryaerophilus MDC1625
A. cryaerophilus MDC1271
A. cryaerophilus A169-B
A. skirrowii MDC1645
A. skirrowii MDC1646
A. skirrowii MDC1736
A. skirrowii MDC1273
A. skirrowii 449-80
A. skirrowii MDC1631
A. thereius MDC1738
A. thereius MDC1739
A. thereius MDC1639
A. trophiarum MDC1749
A. lanthierii AF1440
A. lanthierii AF1430
A. lanthierii AF1581
A. septicus AF1078
A. nitrofigilis MDC1274
A. nitrofigilis CI
A. nitrofigilis DSM-7299
A. nitrofigilis MDC1635
A. nitrofigilis MDC1737
A. nitrofigilis MDC1634
A. sp. F128-2
A. anaerophilus DSM-24636T
A. sp. MDC1641
A. sp. MDC1767
A. molluscorum MDC1719
A. molluscorum MDC1720
A. halophilus MDC1275
A. halophilus DSM-18005
A. marinus MDC1741
A. marinus CL-S1
A. mytili MDC1638
A. mytili MDC1637
A. mytili MDC1636
Helicobacter pylori
0.3

C

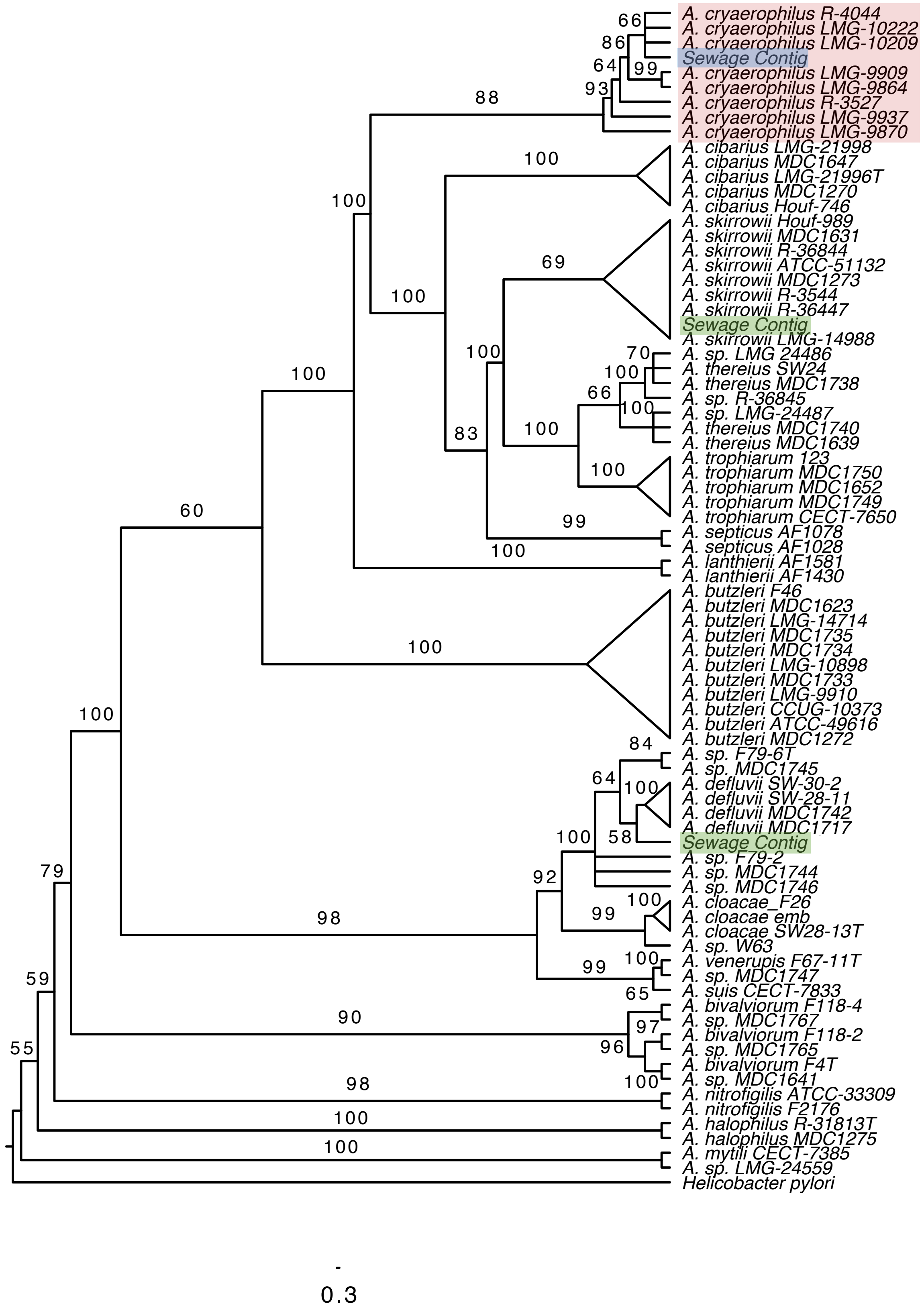
A. cryaerophilus R-4044
A. cryaerophilus LMG-10222
A. cryaerophilus LMG-10209
Sewage Contig
A. cryaerophilus LMG-9909
A. cryaerophilus LMG-9864
A. cryaerophilus R-3527
A. cryaerophilus LMG-9937
A. cryaerophilus LMG-9870
A. cibarius LMG-21998
A. cibarius MDC1647
A. cibarius LMG-21996T
A. cibarius MDC1270
A. cibarius Houf-746
A. skirrowii Houf-989
A. skirrowii MDC1631
A. skirrowii R-36844
A. skirrowii ATCC-51132
A. skirrowii MDC1273
A. skirrowii R-3544
A. skirrowii R-36447
Sewage Contig
A. skirrowii LMG-14988
A. sp. LMG 24486
A. thereius SW24
A. thereius MDC1738
A. sp. R-36845
A. sp. LMG-24487
A. thereius MDC1740
A. thereius MDC1639
A. trophiarum 123
A. trophiarum MDC1750
A. trophiarum MDC1652
A. trophiarum MDC1749
A. trophiarum CECT-7650
A. septicus AF1078
A. septicus AF1028
A. lanthierii AF1581
A. lanthierii AF1430
A. butzleri F46
A. butzleri MDC1623
A. butzleri LMG-14714
A. butzleri MDC1735
A. butzleri MDC1734
A. butzleri LMG-10898
A. butzleri MDC1733
A. butzleri LMG-9910
A. butzleri CCUG-10373
A. butzleri ATCC-49616
A. butzleri MDC1272
A. sp. F79-6T
A. sp. MDC1745
A. defluvii SW-30-2
A. defluvii SW-28-11
A. defluvii MDC1742
A. defluvii MDC1717
Sewage Contig
A. sp. F79-2
A. sp. MDC1744
A. sp. MDC1746
A. cloacae_F26
A. cloacae emb
A. cloacae SW28-13T
A. sp. W63
A. venerupis F67-11T
A. sp. MDC1747
A. suis CECT-7833
A. bivalviorum F118-4
A. sp. MDC1767
A. bivalviorum F118-2
A. sp. MDC1765
A. bivalviorum F4T
A. sp. MDC1641
A. nitrofigilis ATCC-33309
A. nitrofigilis F2176
A. halophilus R-31813T
A. halophilus MDC1275
A. mytili CECT-7385
A. sp. LMG-24559
Helicobacter pylori
0.3

D

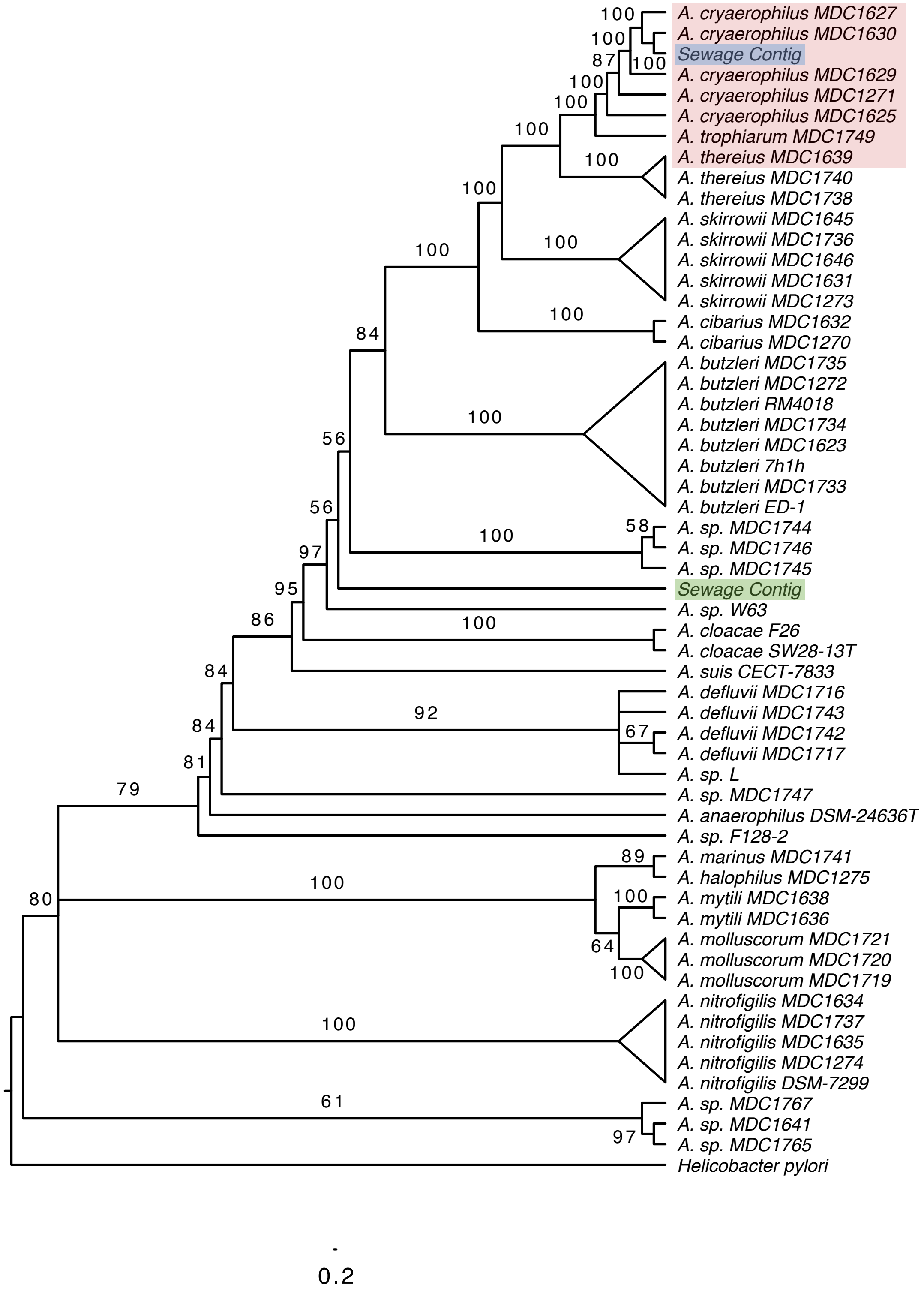
A. cryaerophilus MDC1627
A. cryaerophilus MDC1630
Sewage Contig
A. cryaerophilus MDC1629
A. cryaerophilus MDC1271
A. cryaerophilus MDC1625
A. trophiarum MDC1749
A. thereius MDC1639
A. thereius MDC1740
A. thereius MDC1738
A. skirrowii MDC1645
A. skirrowii MDC1736
A. skirrowii MDC1646
A. skirrowii MDC1631
A. skirrowii MDC1273
A. cibarius MDC1632
A. cibarius MDC1270
A. butzleri MDC1735
A. butzleri MDC1272
A. butzleri RM4018
A. butzleri MDC1734
A. butzleri MDC1623
A. butzleri 7h1h
A. butzleri MDC1733
A. butzleri ED-1
A. sp. MDC1744
A. sp. MDC1746
A. sp. MDC1745
Sewage Contig
A. sp. W63
A. cloacae F26
A. cloacae SW28-13T
A. suis CECT-7833
A. defluvii MDC1716
A. defluvii MDC1743
A. defluvii MDC1742
A. defluvii MDC1717
A. sp. L
A. sp. MDC1747
A. anaerophilus DSM-24636T
A. sp. F128-2
A. marinus MDC1741
A. halophilus MDC1275
A. mytili MDC1638
A. mytili MDC1636
A. molluscorum MDC1721
A. molluscorum MDC1720
A. molluscorum MDC1719
A. nitrofigilis MDC1634
A. nitrofigilis MDC1737
A. nitrofigilis MDC1635
A. nitrofigilis MDC1274
A. nitrofigilis DSM-7299
A. sp. MDC1767
A. sp. MDC1641
A. sp. MDC1765
Helicobacter pylori
100
100
87
100
100
100
100
100
100
100
100
84
100
56
56
100
58
97
95
100
86
84
92
67
84
81
79
89
80
100
100
64
100
100
61
97
0.2

FIG S2. Genome bins found in initial clustering using Albertsen et al. 2013 differential coverage binning procedure for metagenomic data. The top right cluster with ~200x coverage contained all *A. cryaerophilus* housekeeping genes (Fig. S1) and was selected for secondary refinement and finishing.

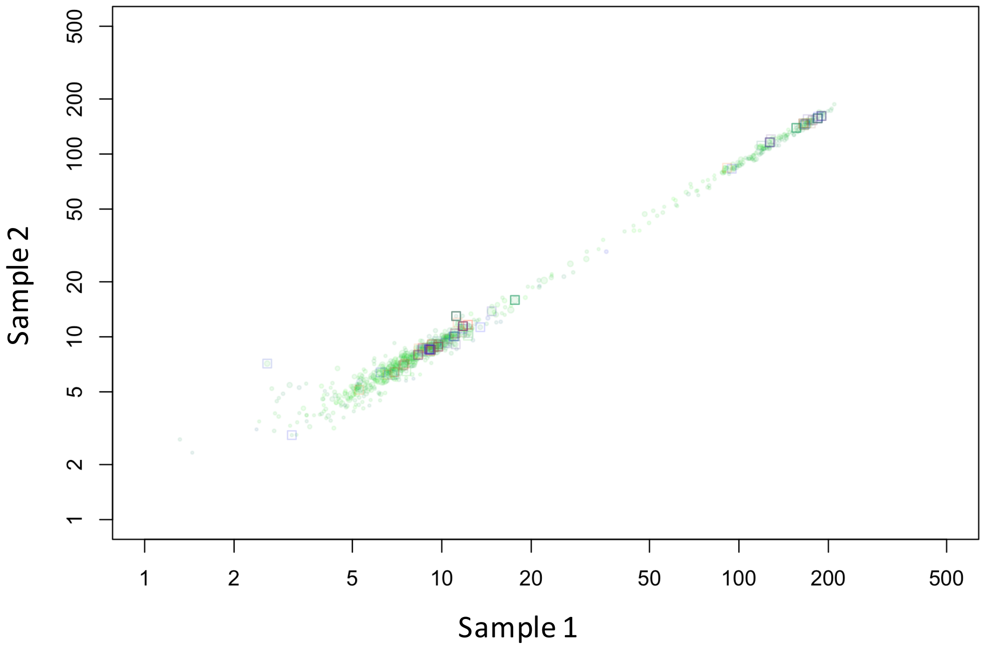

FIG S3. Phylogenetic trees to validate HGTector data. Bayesian trees of *Arcobacter cryaerophilus* ORFs **(A)** scaffold1_44 (tetracycline resistance ribosomal protection protein, 2151nt) and **(B)** scaffold228_1 (chloramphenicol phosphotransferase, 258nt) that place the ORFs outside of the phylum Proteobacteria (in blue).

A

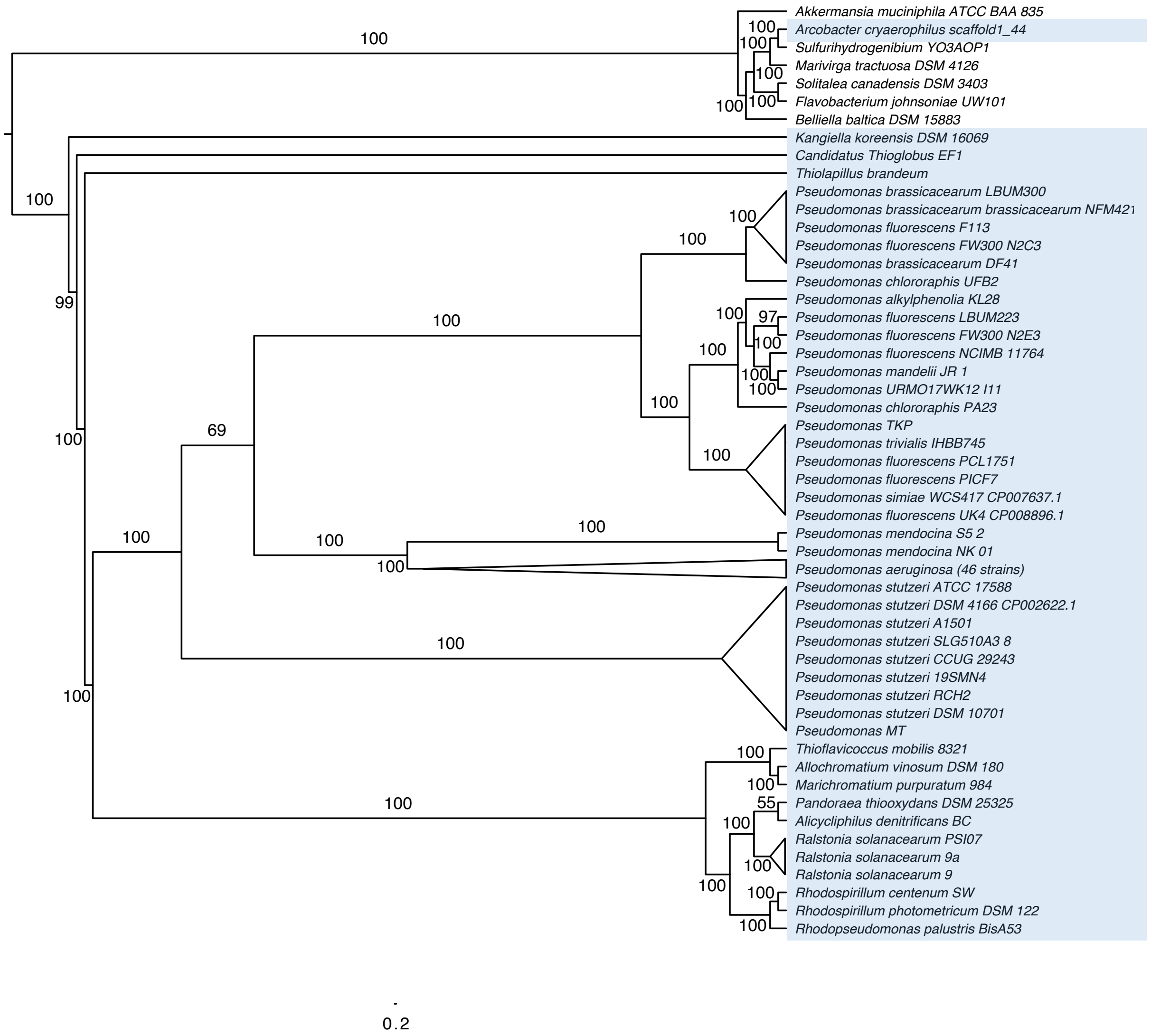

B

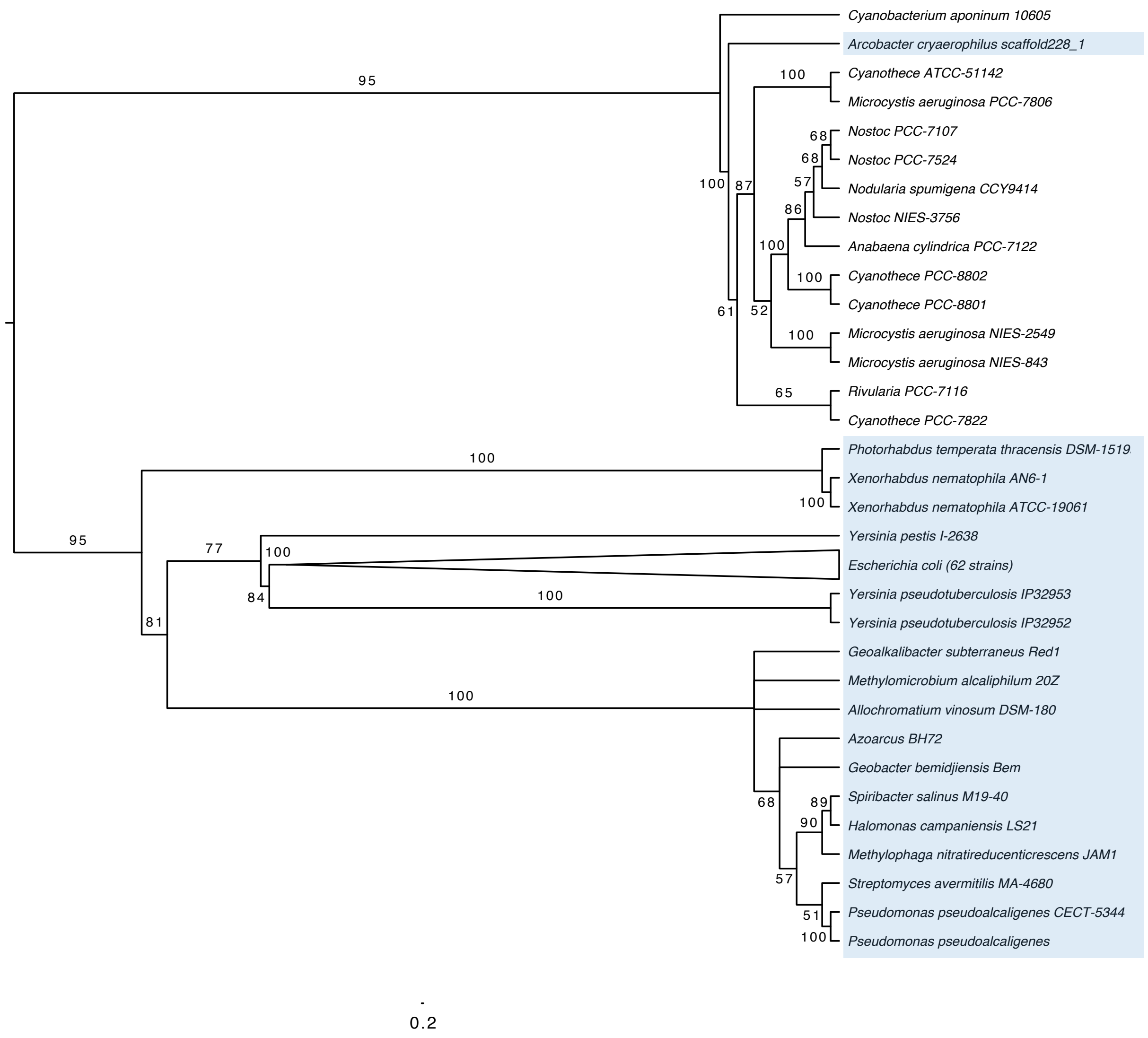
Cyanobacterium aponinum 10605
Arcobacter cryaerophilus scaffold228_1
Cyanothece ATCC-51142
Microcystis aeruginosa PCC-7806
Nostoc PCC-7107
Nostoc PCC-7524
Nodularia spumigena CCY9414
Nostoc NIES-3756
Anabaena cylindrica PCC-7122
Cyanothece PCC-8802
Cyanothece PCC-8801
Microcystis aeruginosa NIES-2549
Microcystis aeruginosa NIES-843
Rivularia PCC-7116
Cyanothece PCC-7822
Photorhabdus temperata thracensis DSM-1519
Xenorhabdus nematophila AN6-1
Xenorhabdus nematophila ATCC-19061
Yersinia pestis I-2638
Escherichia coli (62 strains)
Yersinia pseudotuberculosis IP32953
Yersinia pseudotuberculosis IP32952
Geoalkalibacter subterraneus Red1
Methylomicrobium alcaliphilum 20Z
Allochromatium vinosum DSM-180
Azoarcus BH72
Geobacter bemidjiensis Bem
Spiribacter salinus M19-40
Halomonas campaniensis LS21
Methylophaga nitratireducenticrescens JAM1
Streptomyces avermitilis MA-4680
Pseudomonas pseudoalcaligenes CECT-5344
Pseudomonas pseudoalcaligenes
0.2

FIG S4. Genome alignment of *Arcobacter butzleri* (top) and *Arcobacter acryaerophilus* (bottom). Homologous "locally collinear blocks" are identified using Mauve and connected with lines of the same color to show genome rearrangements.

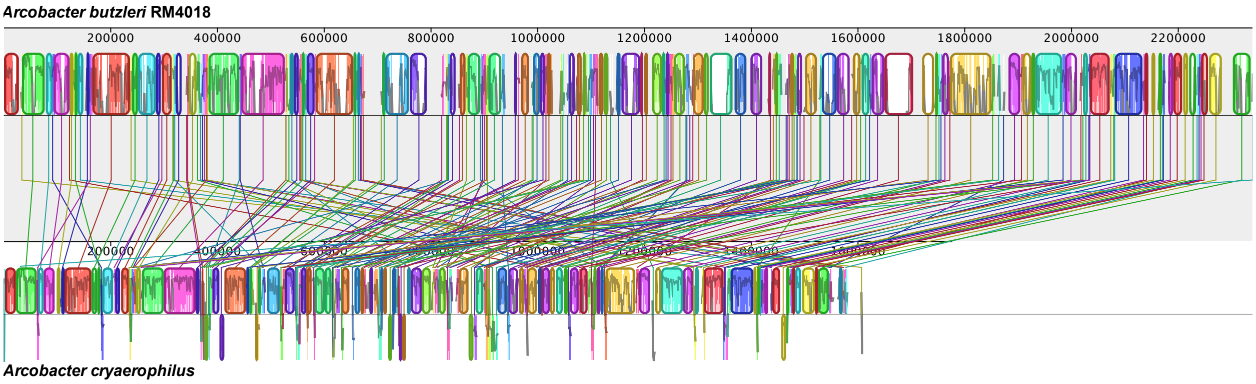

TABLE S1. DNA/RNA Reads and Assembly Totals

	Reads	Sample 1	Sample 2	Sample 3
DNA	Raw	27,149,400	56,951,058	69,948,228
	Cleaned	17,632,699	37,306,119	46,473,784
	Sub Sample	17,000,000	17,000,000	17,000,000
	Mapped to Sewage Contigs	7,703,851	10,257,818	11,710,891
	Mapped to *Arcobacter* Contigs	6,947,969	9,192,258	10,294,926
RNA	Raw	142,205,531	91,010,362	58,196,397
	Cleaned	125,234,635	84,879,463	54,872,132
	Sub Sample	50,000,000	50,000,000	50,000,000
	Mapped to Sewage Contigs	8,641,383	6,985,173	7,355,494
	Mapped to *Arcobacter* Contigs	8,122,362	6,409,562	6,756,931
Contigs	Raw	6,522	15,910	17,210
	Sub Sample	6,000	6,000	6,000
	Average Length	1,189	1,145	1,087
	Length Range	300 - 27,864	300 - 27,810	300 - 18,778
	Phylum Annotated	2,866	3,433	3,264

TABLE S2. Comparison of annotated categories in *Arcobacter*.

Category	Ac[a]	Ab[b]	Category	Ac	Ab
Virulence Factors			Antibiotic Resistance		
Acid resistance	1	1	Aminocoumarin	17	15
Adhesion	18	20	Aminoglycosides	8	23
Anti-apoptosis	1	1	Bacitracin	4	5
Biofilm	1	1	Beta Lactams	11	16
Capsule	1	1	Bicyclomycin	0	2
Cell surface and memb. prot.	5	6	Chloramphenicol	4	4
Cell-to-cell spread	1	1	Ciprofloxacin	4	4
Cellular metabolism	22	12	Elfamycin	0	2
Chaperone	3	1	Erythromycin	3	2
Cording	2	1	Fluoroquinolones	18	21
Defense agnst. host imm. resp.	3	3	Fosfomycin	2	1
Intracellular survival and repl.	30	20	Fosmidomycin	1	2
Invasion	11	14	Glycylcycline	1	0
Iron uptake	4	14	Isoniazid	0	1
Mitogenic	3	1	Kanamycin	0	2
Modulate host immune resp.	3	9	Lincosamide	5	6
Phosphate uptake	3	0	Macrolide	26	25
Regulation of gene expression	4	5	Mupirocin	4	1
Secretion	4	3	Norfloxacin	2	3
Stress	5	3	Penicillin	2	6
Two-component system	6	2	Polymyxin	9	9
Type III secretion	4	1	Roxithromycin	1	0
Type VII secretion	0	1	Streptogramin	9	9
Virulence	153	114	Streptomycin	0	4
Zinc uptake	2	1	Sulfonamide	1	1
			Teicoplanin	0	3
Transposases			Tetracycline	12	24
IS1	26	34	Thiostrepton	1	1
IS110	4	0	Tigecycline	2	6
IS116	0	3	Triclosan	2	3
IS21	4	1	Vancomycin	13	27
IS3	6	4			
IS4-5	0	5	Prophage genes	290	320
IS408	4	0			
IS4like	13	2	Single copy genes	100	106
IS66	4	8			

[a]*A. cryaerophilus*, [b]*A. butzleri*

TABLE S3. Number and expression of antibiotic resistance categories annotated in *A. cryaerophilus*.

Antibiotic Resistance Category	Average Total ORFs	Average Expressed ORFs
Aminocoumarin	17	17.0
Aminoglycosides	8	8.0
Bacitracin	4	4.0
Beta Lactams	11	11.0
Chloramphenicol	4	4.0
Ciprofloxacin	4	4.0
Erythromycin	3	3.0
Fluoroquinolones	18	18.0
Fosfomycin	2	1.7
Fosmidomycin	1	1.0
Glycylcycline	1	1.0
Lincosamide	5	5.0
Macrolide	26	25.7
Mupirocin	4	3.7
Norfloxacin	2	2.0
Penicillin	2	2.0
Polymyxin	9	9.0
Streptogramin	9	9.0
Sulfonamide	1	1.0
Tetracycline	12	12.0
Thiostrepton	1	1.0
Tigecycline	2	2.0
Triclosan	2	2.0
Vancomycin	13	12.7

TABLE S4. Number and expression of antibiotic resistance categories annotated in whole sewage sample.

Antibiotic Resistance Category	Average Total ORFs	Average Expressed ORFs
Acriflavine	29	25.0
Aminocoumarin	275	210.3
Aminoglycosides	184	137.7
Bacitracin	140	90.0
Beta Lactams	323	249.7
Bicyclomycin	13	10.3
Chloramphenicol	148	115.3
Ciprofloxacin	113	77.7
Deoxycholate	2	0.0
Doxorubicin	1	1.0
Elfamycin	52	48.7
Enoxacin	8	1.3
Erythromycin	33	29.7
Fluoramphenicol	3	1.0
Fluoroquinolones	471	346.7
Fosfomycin	22	17.0
Fosmidomycin	19	13.0
Glycylcycline	17	16.0
Isoniazid	20	19.0
Kanamycin	14	6.0
Lincomycin	6	2.7
Lincosamide	168	130.3
Macrolide	682	485.3
Mupirocin	57	51.7
Norfloxacin	44	28.3
Penicillin	54	47.7
Polymyxin	134	108.0
Puromycin	6	4.0
Rifampin	27	18.7
Roxithromycin	4	4.0
Streptogramin	144	112.3
Streptomycin	35	27.7
Sulfonamide	23	15.3
Teicoplanin	88	54.3
Tetracenomycin C	2	1.3
Tetracycline	392	317.3
Tetraphenylarsonium Chloride	6	4.0
Thiostrepton	21	15.3
Tigecycline	55	40.7
Triclosan	43	38.3
Trimethoprim	35	24.0
Vancomycin	244	162.7

TABLE S5. Number and expression of virulence factor categories annotated in *A. cryaerophilus*.

Virulence Factor Category	Average Total ORFs	Average Expressed ORFs
Acid resistance	1	1.0
Adhesion	18	17.7
Anti-apoptosis	1	1.0
Biofilm	1	1.0
Capsule	1	1.0
Cell surface and memb. proteins	5	5.0
Cell-to-cell spread	1	1.0
Cellular metabolism	22	22.0
Chaperone	3	3.0
Cording	2	2.0
Defense against host imm. resp.	3	3.0
Intracell. survival and replication	30	30.0
Invasion	11	11.0
Iron uptake	4	3.7
Mitogenic	3	3.0
Modulate host immune response	3	3.0
Phosphate uptake	3	3.0
Regulation of gene expression	4	4.0
Secretion	4	4.0
Stress	5	5.0
Two-component system	6	6.0
Type III secretion	4	4.0
Virulence	153	151.0
Zinc uptake	2	1.7

TABLE S6. Number and expression of virulence factor categories annotated in whole sewage sample.

Virulence Factor Category	Average Total ORFs	Average Expressed ORFs
Acid resistance	33	28.0
Acquisition of nutrients	2	1.0
Actin-based motility	7	3.0
Adhesion	248	207.3
Anti-apoptosis	19	17.0
Apoptosis	1	1.0
Avoidance of host defense	1	0.0
Bile resistance	1	1.0
Biofilm	16	15.3
Capsule	43	38.7
Cell surface and memb. proteins	82	75.0
Cell wall	1	1.0
Cell-to-cell spread	13	11.0
Cellular metabolism	233	191.0
Chaperone	41	35.3
Cording	45	36.7
Defense against host imm. resp.	90	74.0
Drug efflux system	6	4.7
Escape from the phagosome	8	4.7
Excretion	1	0.0
Heme uptake	5	3.0
Intracell. survival and replication	635	502.3
Invasion	347	291.3
Iron uptake	81	54.0
LPS (Lipopolysaccharide)	4	3.7
Mitogenic	29	29.0
Modulate host immune response	78	60.0
Molecular mimicry	10	8.0
Protease	5	3.0
Regulation of gene expression	46	35.7
Secretion	93	81.3
Stress	67	50.0
Toxin	18	10.7
Two-component system	83	57.7
Type III secretion	53	51.3
Type VII secretion	13	8.7
Virulence	2965	2294.7

DATA SET S1. Expression of ORFs mapped to antibiotic resistance categories

DATA SET S2. Comparison of overlapping genes and annotations in *Arcobacter*

DATA SET S3. Comparison of *Arcobacter* in RAST

DATA SET S4. Expression of ORFs mapped to virulence factors

DATA SET S5. Descriptive statistics of *A. cryaerophilus* contigs
